# Activity Quantification of Fuel Cell Catalysts via Sequential Poisoning by Multiple Reaction Inhibitors

**DOI:** 10.3390/nano12213800

**Published:** 2022-10-28

**Authors:** Yunjin Kim, Jiho Min, Keonwoo Ko, Bathinapatla Sravani, Sourabh S. Chougule, Yoonseong Choi, Hyeonwoo Choi, SeoYeong Hong, Namgee Jung

**Affiliations:** Graduate School of Energy Science and Technology (GEST), Chungnam National University, 99 Daehak−ro, Yuseong−gu, Daejeon 34134, Korea

**Keywords:** fuel cells, oxygen reduction reaction, multiple reaction inhibitor, activity quantification, active site, poisoning

## Abstract

The development of non−Pt or carbon−based catalysts for anion exchange membrane fuel cells (AEMFCs) requires identification of the active sites of the catalyst. Since not only metals but also carbon materials exhibit oxygen reduction reaction (ORR) activity in alkaline conditions, the contribution of carbon-based materials to ORR performance should also be thoroughly analyzed. However, the conventional CN^−^ poisoning experiments, which are mainly used to explain the main active site of M−N−C catalysts, are limited to only qualitative discussions, having the potential to make fundamental errors. Here, we report a modified electrochemical analysis to quantitatively investigate the contribution of the metal and carbon active sites to ORR currents at a fixed potential by sequentially performing chronoamperometry with two reaction inhibitors, CN^−^ and benzyl trimethylammonium (BTMA^+^). As a result, we discover how to quantify the individual contributions of two active sites (Pt nanoparticles and carbon support) of carbon−supported Pt (Pt/C) nanoparticles as a model catalyst. This study is expected to provide important clues for the active site analysis of carbon-supported non−Pt catalysts, such as M−N−C catalysts composed of heterogeneous elements.

## 1. Introduction

As climate change due to the greenhouse effect caused by carbon dioxide (CO_2_) becomes more serious around the world, renewable energy resources to replace fossil fuels are attracting a lot of attention. Among various renewable energy resources, hydrogen is recognized as an eco−friendly and sustainable energy resource. Accordingly, in recent years, hydrogen fuel cells have been intensively developed as promising energy conversion devices that can replace internal combustion engines [1,2,3,4,5].

In hydrogen fuel cell systems, carbon-supported Pt (Pt/C) nanoparticles are the most widely used as representative electrocatalysts, and simultaneously serve as a performance evaluation standard in the development of novel catalysts [6,7,8]. However, since Pt is a precious and expensive material, it cannot be a reasonable final candidate when considering the commercialization of fuel cells. Therefore, extensive studies of various catalyst structures have been performed to ensure high oxygen reduction reaction (ORR) activity while lowering the catalyst cost. For instance, alloy catalysts with reduced Pt usage, non−Pt catalysts using other transition metals, and carbon-based materials that do not even contain metals have been developed [9,10,11,12,13,14].

Meanwhile, in the study of applying a catalyst that replaces Pt in fuel cells, a clear understanding of the reaction mechanism is required, and the identification of the active site of the catalyst must precede. In this context, experimental results describing the reaction mechanisms and active sites of non−Pt or carbon-based catalysts have been reported for decades [15,16,17,18,19]. For instance, the atomically dispersed transition metal (M = Fe, Co, Mn, etc.) and nitrogen co-doped carbon (M−N−C) catalysts have been proposed as the most promising alternative to Pt-based catalysts [20,21]. In general, the main active site of the M−N−C catalysts is elucidated through the experimental method of adding a small amount of a reaction inhibitor, such as Cl^−^, ClO_4_^−^, NO_2_^−^, NO_3_^−^, CN^−^, and tris(hydroxymethyl)aminomethane, to the electrolyte [22,23,24]. One can observe a change in the i–V curve for ORR since the reaction inhibitors poison the catalyst due to the strong binding energy to the metal surface.

However, most of these studies are limited to the qualitative discussions that the center Fe (or Co) atoms will become the main active sites through the reduced ORR performance after poisoning. In particular, there is a possibility of making an error of assuming that the main active site is completely poisoned by the reaction inhibitors, despite the relatively low but clear ORR activity observed within a wide potential range. For instance, it is well known that the binding energy of CN^−^ to the center metal atom of the Fe−N−C catalyst is much smaller than that to the surface of Pt nanoparticles, making it impossible to completely poison the Fe−N−C catalyst by CN^−^. In addition, since carbon materials exhibit relatively high activity in alkaline solutions, the conventional electrochemical analysis using only CN^−^ may reveal fundamental limitations in an alkaline electrolyte for the half-cell test of anion exchange membrane fuel cells (AEMFCs) [25,26]. On the other hand, D. Malko et al. demonstrated a protocol to determine the number of metal active sites of the M−N−C catalysts via nitrite adsorption followed by reductive stripping [27]. However, the electrochemical analysis was also limited to the understanding of the center metal atoms except for the active carbons. Accordingly, a quantitative analysis of the contribution of each component (metal or carbon) to ORR performance at a given potential has not been conducted in most previous studies [23,28,29,30].

In this work, based on the CN^−^ poisoning test, we propose a modified electrochemical method to quantitatively identify the contribution of elemental components of electrocatalysts to ORR activity in alkaline solution by additionally introducing a reaction inhibitor, benzyl trimethylammonium (BTMA^+^). A commercial Pt/C catalyst and Vulcan XC−72 carbon are used as a simple model system, respectively, to demonstrate the effectiveness of the developed quantitative analysis. At first, a catalyst poisoning experiment using each reaction inhibitor is performed at a fixed potential to observe changes in the electrochemical property according to the catalyst structure and components. Then, during ORR, the two reaction inhibitors are sequentially introduced at the corresponding potential, and the individual contributions of the Pt and carbon support constituting the model catalyst, Pt/C, are quantitatively analyzed. This study is expected to provide important clues for the analysis of the active sites of non−Pt catalysts composed of various heterogeneous elements such as M−N−C catalysts.

## 2. Materials and Methods

To confirm the physical characterization of commercial Pt/C (20 wt%, Premetek, Cherry Hill, NJ, USA) catalyst and carbon support (Vulcan XC-72, Cabot, Boston, MA, USA), transmission electron microscopy (TEM) (HF5000, Hitachi, Tokyo, Japan) and powder X-ray diffraction (XRD) (D8 ADVANCE, Bruker, Billerica, MA, USA) were used. All electrochemical measurements were conducted at room temperature (25 °C) using a standard three−compartment electrochemical cell with a rotating disk electrode (RDE). The three−electrode cell consists of a Ag/AgCl electrode in saturated KCl (3 M) aqueous solution, a Pt sheet, and glassy carbon (GC) electrode with a geometric area of 0.196 cm^2^ as the reference electrode, counter electrode, and working electrode, respectively. The potentials measured against a Ag/AgCl electrode were converted to the potentials versus the reversible hydrogen electrode (RHE). Pt/C catalyst ink was prepared by mixing 5 mg of catalyst, 34.4 μL of Nafion solution (Sigma−Aldrich, Burlington, MA, USA), 50 μL of DI water, and 450 μL 2−propanol by sonication for 15 min. In the same way, the Vulcan XC−72 ink was prepared with 4 mg of Vulcan, the same amount of Nafion solution, and 500 μL of 2-propanol. Then, 5 μL of the catalyst ink was loaded onto the GC of RDE and dried at room temperature. In the case of Pt/C catalyst, the total catalyst loading was 238.7 μg cm^−2^ (Pt loading: 47.8 μg cm^−2^), and the Vulcan XC−72 sample loading was 190.9 μg cm^−2^, which is equal to the amount of the carbon support in the Pt/C catalyst loading. The chronoamperometry was performed at 0.7 V with rotation of the RDE at 1600 rpm in O_2_-saturated alkaline electrolytes. The electrolyte used in each experiment was 0.1 M KOH, 0.01 M KCN−mixed 0.1 M KOH, and 0.1 M BTMAOH which is an alkaline electrolyte with a benzene group. In addition, the performance recovery experiment was conducted by washing the GC with DI water several times. The chronoamperometry currents over time were normalized to the initial ORR currents of each sample.

## 3. Results and Discussion

**Figure 1:**
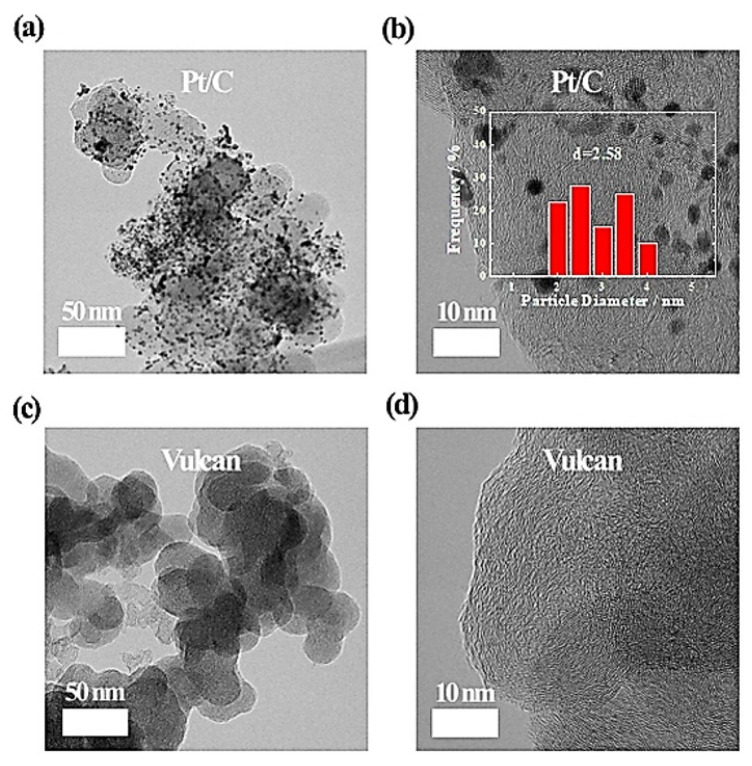
TEM images of (**a**,**b**) Pt/C and (**c**,**d**) Vulcan XC−72. The inset of (**b**) shows the size distribution of Pt nanoparticles of Pt/C.

**Figure 2:**
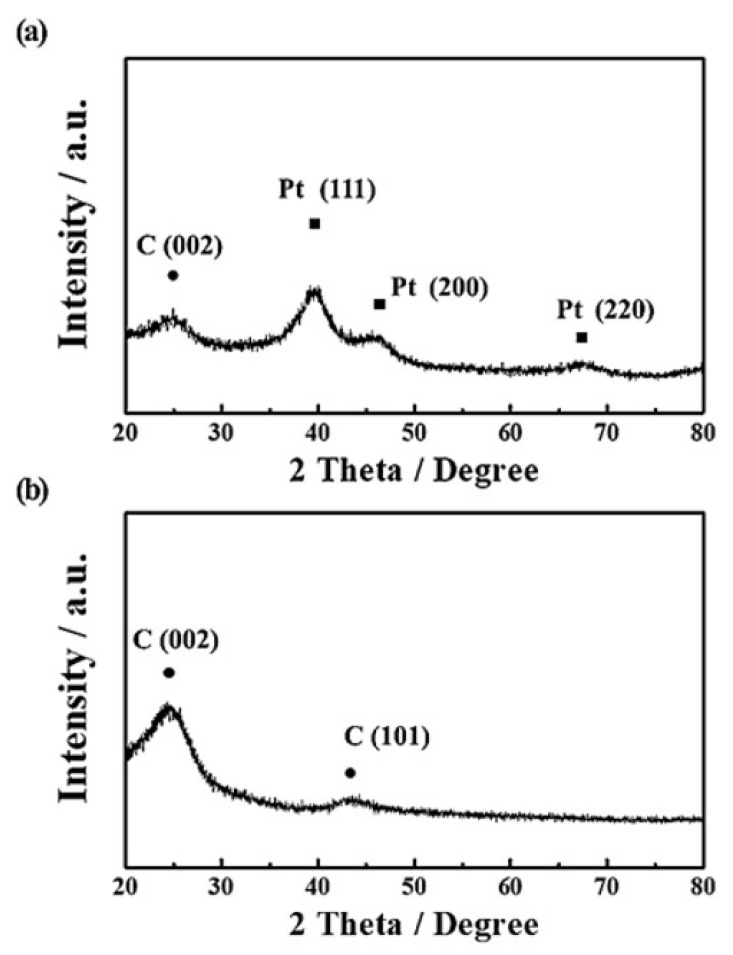
XRD patterns of (**a**) Pt/C and (**b**) Vulcan XC−72.

The physical structures of Pt/C catalyst and Vulcan XC−72 carbon were characterized by using TEM and XRD, as shown in Figure 1 and Figure 2. The TEM images in Figure 1a,b reveal that the Pt/C catalyst has very small Pt nanoparticles of ~2.58 nm uniformly dispersed on the carbon support. On the other hand, in the case of the Vulcan XC−72 sample, carbon particles of 30–50 nm, similar to the size of the carbon support of Pt/C, are observed without metal nanoparticles (Figure 1c,d). In addition, as shown in Figure 2, the XRD peaks of the Pt/C catalyst show the Pt(111), Pt(200), and Pt(220) planes along with the C(002) plane, whereas the Vulcan XC−72 sample indicates only the C(002) and C(101) peaks due to the absence of metal particles [31,32,33,34]. Accordingly, it is confirmed that the Pt/C catalyst is composed of Pt nanoparticles and carbon support, and the Vulcan XC−72 sample consists only of carbon particles.

Chronoamperometry was then performed under various operating conditions with different electrolytes to electrochemically analyze the contribution of each component, Pt nanoparticle and carbon support, to ORR activity of the Pt/C catalyst. First, 0.1 M KOH was selected as a commonly used alkaline electrolyte to observe the inevitable degradation of ORR performance for Pt/C and Vulcan XC−72 samples over time. Second, 0.01 M KCN−mixed 0.1 M KOH solution was used to investigate the effect of CN^−^ on the Pt nano-particle and carbon support. Third, chronoamperometry was carried out in 0.1 M BTMAOH solution, which can also deteriorate the ORR performance of the catalyst due to the adsorption capacity of benzene by electrostatic or π−p interactions with the carbon surface [35,36,37].

**Figure 3:**
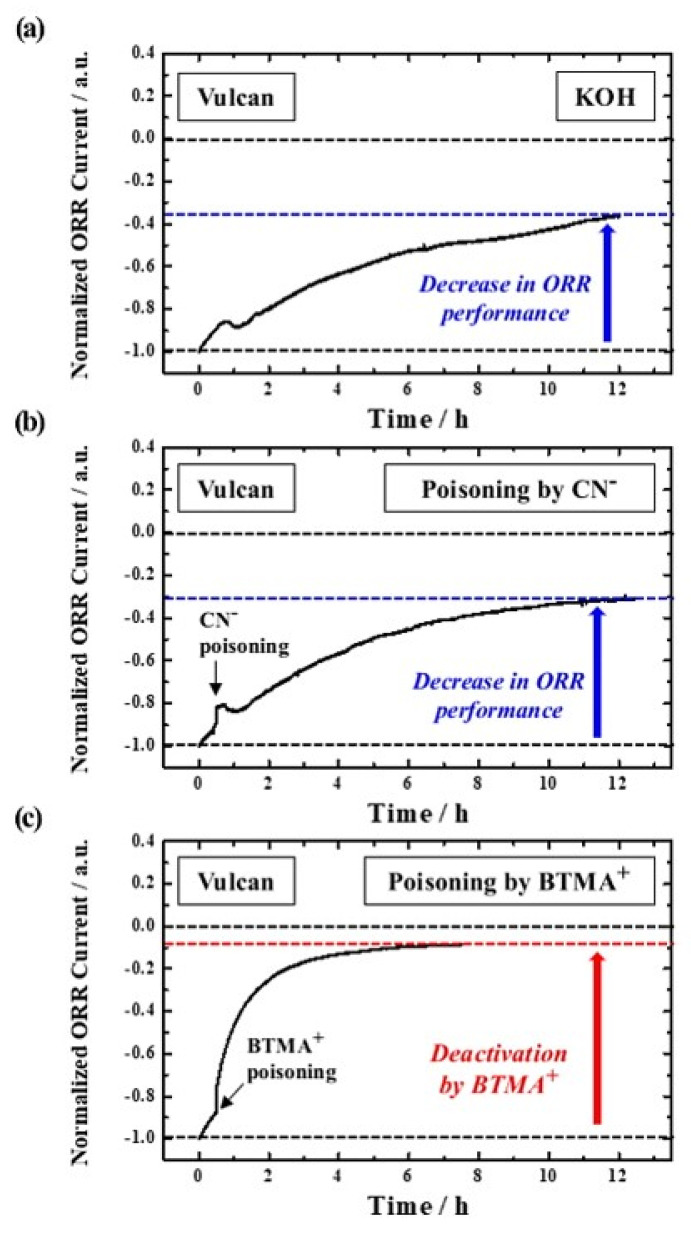
Chronoamperograms of Vulcan XC−72 at 0.7 V in O_2_−saturated (**a**) 0.1 M KOH, (**b**) 0.01 M KCN−mixed 0.1 M KOH, and (**c**) 0.1 M BTMAOH.

Figure 3a–c show the change in ORR performance of the Vulcan XC−72 sample under the above-mentioned experimental conditions. First of all, the normalized ORR current of Vulcan XC-72 gradually decreases by ~65% over 12 h in 0.1 M KOH (Figure 3a). Likewise, the performance is similarly reduced by 70% due to the effect of CN^−^, implying that CN^−^ has a negligible poisoning effect on pure carbon material (Figure 3b). As a result, the decrease in ORR performance in both electrolytes is thought to occur naturally and is not due to any poisoning mechanism. In sharp contrast, when BTMA^+^ is used as a reaction inhibitor, the ORR activity of Vulcan XC−72 rapidly decreases by ~92% after 8 h, as shown in Figure 3c. Therefore, it is revealed that BTMA^+^ is the most suitable reaction inhibitor to poison the carbon sample, which is elucidated by the fact that benzene-based molecules such as BTMA^+^ can be electrochemically and/or physically captured by activated carbons.

**Figure 4:**
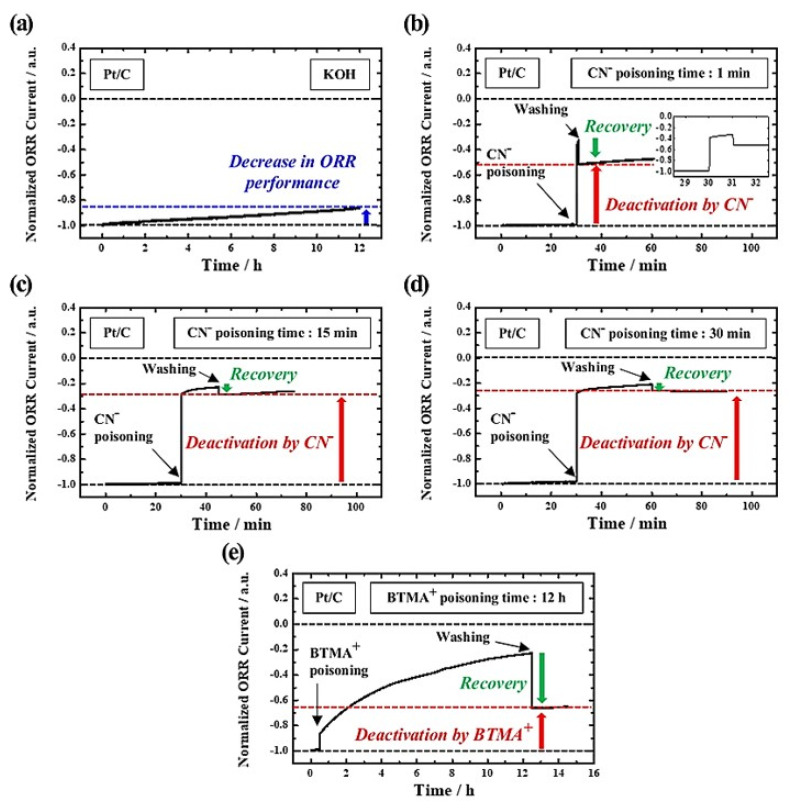
Chronoamperograms of Pt/C at 0.7 V in O_2_−saturated (**a**) 0.1 M KOH, (**b**–**d**) 0.01 M KCN−mixed 0.1 M KOH, and (**e**) 0.1 M BTMAOH. The inset of (**b**) shows an enlarged chronoamperogram of the corresponding data.

Now, we need to focus on the electrochemical test results of Pt/C catalyst. As shown in Figure 4a, it is confirmed that the ORR performance of Pt/C is not significantly reduced even after 12 h in 0.1 M KOH, unlike the Vulcan XC−72 sample. However, it is well known that Pt nanoparticles are rapidly poisoned by CN^−^ due to the strong binding energy [10,38,39]. To investigate the effect of CN^−^ poisoning time on ORR activity of Pt/C, a performance recovery test was conducted by washing the GC with DI water after CN^−^ poisoning for a period of time (1, 15, and 30 min), as shown in Figure 4b–d. After CN^−^ poisoning for 1 min, the normalized ORR current is reduced by ~70%, but immediately returns to ~50% of its initial performance, which implies that Pt nanoparticles cannot be completely poisoned in a short time. As expected, with increasing the poisoning time from 15 to 30 min, the ORR current recovered after the washing step became negligible. Furthermore, the current was reduced by 75~80%, which may be due to complete poisoning of the Pt nanoparticles. Considering these results, we recognized that 30 min is sufficient time to poison the metal nanoparticles.

However, as shown in Figure 4e, when BTMA^+^ is only used as a reaction inhibitor, the normalized ORR current of Pt/C decreases by ~80% after 12 h. Interestingly, after washing, the activity is recovered to ~65% of its initial current, which can be explained by the adsorption of BTMA^+^ not only to the carbon support but also to the Pt nanoparticles. Despite this, since the BTMA^+^ binding energy to the Pt nanoparticle might be weaker than that to the carbon support, we guess the ORR current can be significantly recovered after washing [40,41,42]. Therefore, it is concluded that BTMA^+^ is only suitable for suppressing the activity of carbon materials, whereas CN^−^ is suitable as a reaction inhibitor for metal nanoparticles.

**Figure 5:**
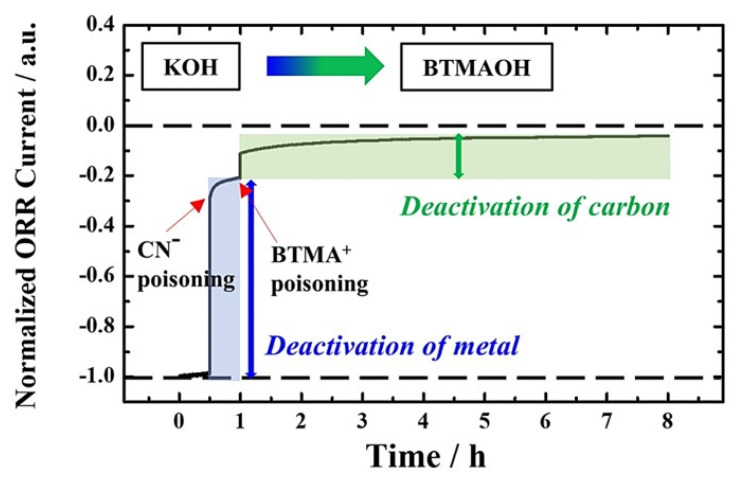
Chronoamperogram of Pt/C at 0.7 V sequentially measured in O_2_−saturated 0.1 M KOH for 30 min, in O_2_−saturated 0.01 M KCN−mixed 0.1 M KOH for 30 min, and in O_2_−saturated 0.1 M BTMAOH for 7 h. When changing the electrolyte to BTMAOH in the last step, the GC was washed several times with DI water to completely remove non−poisoned CN^−^.

Finally, the quantitative analysis of the contribution of metal nanoparticles and carbon support to the ORR current at a fixed potential (0.7 V) was conducted more precisely by sequential introduction of the two inhibitors. As shown in Figure 5, CN^−^ poisoning for 30 min was first carried out in O_2_−saturated 0.1 M KOH solution to deteriorate the ORR activity of Pt only. After washing, the chronoamperometry was continued in 0.1 M BTMAOH solution without CN^−^, resulting in the almost complete suppression of the ORR current.

Consequently, it was unambiguously revealed that the ORR current reduced by ~80% due to CN^−^ poisoning was attributed mainly to the Pt nanoparticles and the remaining 20% of the performance was due to the carbon support. In other words, for the Pt/C catalyst, the contribution of the Pt nanoparticles to ORR performance at 0.7 V is ~4 times higher than that of the carbon support, implying that the main active site is the surface of the Pt nanoparticles as reported elsewhere [43,44,45].

## 4. Conclusions

In summary, through the sequential poisoning by CN^−^ and BTMA^+^, we quantitatively analyzed the contributions of the Pt nanoparticles and carbon support to ORR performance during chronoamperometry at a given potential in alkaline electrolytes. First of all, it was clearly confirmed that CN^−^ and BTMA^+^ are suitable inhibitors for deteriorating the Pt nanoparticles and carbon support, respectively. Then, by sequentially introducing the two reaction inhibitors (CN^−^ → BTMA^+^) during the chronoamperometry, we discovered that the contribution of the Pt nanoparticles to the ORR current at 0.7 V was ~80%, whereas that of the carbon support accounts for ~20%. In other words, by precisely quantifying the activity contributions of the two active sites, the surface of the Pt nanoparticles was identified as the main active site for ORR. From this point of view, we believe that the ORR activity of M−N−C catalysts, which consist of metal atom−decorated carbon materials, can also be analyzed using a sequential poisoning with multiple reaction inhibitors. Based on the CN^−^ and BTMA^+^ poisoning tests for Pt/C and Vulcan XC−72, we will continue to develop electrochemical methods to find the key factors affecting the ORR performance of M−N−C catalysts, providing insight into the design of non-Pt catalysts for AEMFCs.

## Data Availability

No data were used for the research described in the article.
